# Gains in Grain Yield of Extra-Early Maize during Three Breeding Periods under Drought and Rainfed Conditions

**DOI:** 10.2135/cropsci2018.03.0168

**Published:** 2018-08-30

**Authors:** B. Badu-Apraku, A. O. Talabi, B. E. Ifie, Y. C. Chabi, K. Obeng-Antwi, A. Haruna, R. Asiedu

**Affiliations:** 1 International Institute of Tropical Agriculture, Ibadan, Nigeria; 2 West Africa Centre for Crop Improvement, Univ. of Ghana, Accra; 3 Maize Improvement, INRAB, Cotonou, Benin; 4 Maize Improvement Unit, CRI, Ghana; 5 Savanna Agricultural Research Institute, Tamale, Ghana

## Abstract

Drought is a key maize (*Zea mays* L.) production constraint in sub-Saharan Africa. Fourteen, fifteen, and twenty-five extra-early maturing maize cultivars, with varying *Striga* resistance and drought and low soil N tolerance, were developed from 1995 to 2000 (Period 1), 2001 to 2006 (Period 2), and 2007 to 2012 (Period 3), respectively. The objectives of this study were to examine yield gains in the cultivars and to investigate inter-trait relationships and yield stability under six drought and 17 rainfed conditions in West Africa from 2013 to 2016. Annual rate of yield increase across cultivars was 0.034 (3.28%) and 0.068 Mg ha^−1^ (2.25%), whereas yield gains per period were 0.17 and 0.38 Mg ha^−1^ under drought and rainfed environments, respectively. Yield gains under drought and rainfed environments were related to prolonged flowering period, increased plant and ear heights, improved stalk lodging, and ear and plant aspects, whereas delayed leaf senescence and increased number of ears per plant accompanied yield improvement under drought only. Ear aspect and number of ears per plant were primary contributors to yield and could be used as selection criteria for yield enhancement under drought and rainfed conditions. High-yielding and stable cultivars across all environments based on additive main effects and multiplicative interaction (AMMI) biplot included ‘2004 TZEE-Y Pop STR C_4_’ and ‘TZEE-W Pop STR BC_2_ C_0_’ of Period 2 and ‘2009 TZEE-W STR’, ‘TZEE-Y STR 106’, ‘TZEE-W STR 107’, and ‘TZEE-W DT C_0_ STR C_5_’ of Period 3. These cultivars could be commercialized to improve food self-sufficiency in sub-Saharan Africa.

**MAIZE** (*Zea mays* L.) is a major staple crop in West and Central Africa (WCA). The development and commercialization of extra-early maize that matures in 80 to 85 d have made it possible for maize to spread into the savannas of WCA. This has resulted in the expansion of the crop and rapid replacement of the traditional crops, including the indigenous sorghum [*Sorghum bicolor* (L.) Moench] and pearl millet [*Pennisetum glaucum* (L.) R. Br.], particularly in the savannas of WCA. This is attributable to the fact that extra-early maize cultivars respond better to application of fertilizer, have a shorter growing cycle, and are ready for harvest much earlier than the indigenous sorghum and millet crops. In addition, as a result of the dry spell usually experienced from November of each year to March of the following year, early and extra-early crops are preferred to reduce the hunger gap in July of each year because of the shorter maturity period of the crop. An important factor constraining maize production in the savanna agroecology is drought, which accounts for huge yield losses annually in sub-Saharan Africa (SSA). Global warming, which is usually associated with irregular rainfall patterns, calls for an urgent and effective genetic intervention to increase grain yield and tolerance to drought stress (Badu-Apraku and Fakorede, 2017).

Drought stress and poor soil fertility of tropical soils, especially N, compounds the effects of *Striga hermonthica* on maize because of enhanced secretion of strigolactones, plant hormones that stimulate the germination of *Striga* seeds (Cechin and Press, 1993; Mumera and Below, 1993; Kim and Adetimirin, 1995). Therefore, it is of critical importance to introgress genes for drought tolerance into *Striga*-resistant cultivars in the Guinea and Sudan savannas, which frequently experience intermittent drought stress and low soil fertility. It is therefore not surprising that farmers, who cultivate maize in *Striga*-endemic agroecologies of WCA, prefer cultivars with combined *Striga* resistance and drought tolerance. The WCA farmers are reluctant to accept maize cultivars that are susceptible to both drought stress and *Striga* infestation (Badu-Apraku and Fakorede, 2013).

To facilitate the development of drought-tolerant cultivars and improved technologies targeted at the different agroclimatic conditions in SSA, particularly drought stress, a program was designed specifically to capitalize on the inherent mechanisms for drought escape and drought tolerance in maize and the prevailing production conditions in WCA. The cultivars possessing drought escape mechanisms usually complete critical physiological processes of the life cycle before the onset of drought. This is highly desirable in cultivars developed for farmers in agroecologies prone to terminal drought stress in WCA. On the other hand, drought tolerance is a physiological mechanism in plants, which is genetically controlled and can enable plants to minimize or withstand the adverse effects of drought. Drought-tolerant cultivars are especially invaluable in environments where the occurrence of drought is unpredictable during crop growth and development in WCA. Two approaches have been adopted since 1995 for developing extra-early maize cultivars with enhanced drought tolerance for droughtprone agroecologies of WCA. The first one involves the development of extra-early cultivars that mature before the onset of severe drought. The second strategy involves the development of drought-tolerant cultivars under induced drought stress. Breeding for extra-early-maturing cultivars has been performed in the savanna agroecologies and many cultivars have been developed, released, and commercialized after extensive testing in the diverse agroclimatic conditions of WCA. Since 2007, an important strategy of the IITA Maize Program has been to evaluate extra-early maize inbred lines from diverse sources for drought tolerance. Selected outstanding drought-tolerant inbred lines are also screened for *Striga* resistance under artificial infestation. The outstanding inbred lines possessing both drought tolerance and *Striga* resistance are used to develop hybrids that are then evaluated for adaptation to drought prone and *Striga*-endemic locations. The selected lines have served as invaluable sources of drought tolerance alleles for genetic enhancement of two source populations of extra-early maturity that are being improved using the S_1_ family recurrent selection scheme. Genetic enhancement of the extra-early source populations under managed drought stress using the S_1_ recurrent selection method has generated new productive cultivars possessing alleles for both drought tolerance and *Striga* resistance. The selection for enhanced resistance to *Striga* and improved grain yield performed under low N has resulted in extra-early maize with increased tolerance to low N (Badu-Apraku et al., 2009).

Studies conducted in temperate countries have been used to document breeding progress by comparing the performance of released cultivars developed during different eras in environments similar to those of the tropical regions (Russell, 1984; Voldeng et al., 1997; Specht et al., 1999). For example, Russell (1984) documented genetic gain in grain yield of 0.68% yr^−1^ for cultivars developed in the United States between 1930s and 1980s. Much higher yield gains of 1.7% yr^−1^ were reported by Tollenaar (1989) for outstanding maize hybrids developed between the late 1950s and late 1980s and evaluated under drought conditions in Canada. However, only a few reports are available on yield gains for tropical maize evaluated under drought stress. For example, Masuka et al. (2017b) demonstrated annual gains in grain yield of 0.029, 0.085, 0.11, and 0.193 Mg ha^−1^ for early-maturing open-pollinated varieties (OPVs) under natural drought, low N, optimal conditions, and infestation of the *Maize streak virus* (MSV), respectively, in Eastern and Southern Africa (ESA). Genetic gains under random drought, low-N, rainfed, and MSV-infested conditions for the intermediate-late-maturing cultivars were reported to be 0.042, 0.053, 0.079, and 0.109 Mg ha^−1^ yr^−1^, respectively (Masuka et al., 2017b). However, the authors did not observe any significant gains in grain yield of both early-maturing and late-intermediate-maturing cultivars under managed drought conditions. Annual genetic gains for grain yield of maize hybrids developed by CIMMYT in ESA during the 2000 to 2010 period and evaluated under managed drought stress, random drought, low N, optimal, and MSV-infested conditions were estimated to be 0.325, 0.227, 0.209, 0.109, and 0.141 Mg ha^−1^, respectively (Masuka et al., 2017a). In contrast, studies on genetic gains have been conducted for only OPVs in WCA. For example, Kamara et al. (2004) conducted a study to examine genetic gains from selection of maize cultivars of late maturity, released between 1970 and 1999, in the savannas of Nigeria and reported an annual genetic gain in grain yield of 0.41%. The increase was attributed to higher total biomass production and kernel weight, accompanied by reduction in days to flowering and plant height (PHT). Bello et al. (2014) conducted a comparative study on the response of six maize hybrids, two each from the 1980, 1990, and 2000 eras under three N levels (0, 30, and 90 kg N ha^−1^); the N levels were used as main plots and the six hybrids as subplots. Results revealed that mean grain yield increased by 48.4 and 62.4%, as N increased from 0 to 30 kg ha^−1^ and from 30 to 90 kg ha^−1^, respectively (Bello et al., 2014). The genetic gains in grain yield of 42% (between 1980 and 2000) and of 9% (between 1990 and 2000) were obtained under optimal N fertilization (90 kg of N ha^−1^). The two hybrids of the 2000 era were outstanding in all the agronomic traits and leaf chlorophyll concentration at all N levels. It was concluded that improving traits associated with fertilizer N response could accelerate rate of genetic gains in maize hybrid yields. In another study conducted by Badu-Apraku et al. (2017a), genetic gains in grain yield of 56 extra-early open-pollinated maize cultivars developed during three breeding eras (1995–2000, 2001–2006, and 2007–2012) were estimated under low N and high soil N in Nigeria in 2013 and 2014. They reported genetic gains in grain yield of 0.314 Mg ha^−1^ era^−1^ (13.29%) under low N and 0.493 Mg ha^−1^ era^−1^ (16.84%) under high N. In a similar study conducted between 1988 and 2010 under induced drought stress and optimal (stress-free) growing conditions, Badu-Apraku et al. (2013a) showed that the annual yield gains for early-maturing OPVs were 0.040 and 0.014 Mg ha^−1^ under optimal conditions and induced drought, respectively. Genetic gains in yield of the cultivars tested under drought conditions were accompanied by improved plant aspect (PASP) and husk cover (HUSK), whereas under optimal conditions, yield gains were associated with improved PASP and ear aspect (EASP), increased number of ears per plant (EPP), increase in PHT and ear height (EHT), and improved HUSK. Badu-Apraku et al. (2015a) also evaluated maize cultivars of early maturity under low-N conditions in WCA and reported an increase in grain yield of 0.165 Mg ha^−1^ era^−1^, and a yield range of 2.28 to 2.61 Mg ha^−1^ for the first era (1955–2000) to the third era (2007–2012) cultivars, respectively. Despite the results of these studies, there is complete lack of information on yield gains and changes in other agronomic traits of extra-early-maturing cultivars of the three breeding periods under drought stress and optimal growing conditions. Furthermore, information on trait association during the different breeding periods is crucial for identifying valuable traits and different breeding strategies for enhancing progress in improving extra-early maize for stress tolerance (Badu-Apraku et al., 2015b). The current study was therefore conducted to (i) assess yield gains in extra-early maize cultivars of the three breeding periods (1995–2000 = Period 1, 2001–2006 = Period 2, and 2007–2012 = Period 3) under drought and rainfed environments, (ii) investigate trait associations during the three breeding periods, and (iii) assess the performance of the cultivars relative to grain yield and stability across target research environments.

## MATERIALS AND METHODS

### Development of Extra-Early Cultivars Possessing Mechanisms for Drought Escape and Tolerance to Drought, *Striga*, and Streak Virus

The extra-early populations used for the extraction of inbred lines and cultivars were derived from crosses involving superior accessions, including introduced germplasm selected after extensive testing in WCA (Badu-Apraku and Fakorede, 2001; Badu-Apraku et al., 2007). For about two decades, the S_1_ family selection scheme, artificial *S. hermonthica* field infestation, and screening under managed and random drought have been used by IITA maize scientists to develop one each of white (TZEE-W Pop STR) and yellow (TZEE-Y Pop STR) source populations of extra-early maturity. After genetic enhancement of these populations, a large number of cultivars and inbred lines of extra-early maturity, combining drought tolerance and resistance to *S. hermonthica* and MSV, were extracted from each population. Several extra-early inbred lines in the IITA Maize Program possessed drought escape mechanism(s) and drought tolerance genes. It was therefore expected that the inbred lines would withstand the drought stress occurring during flowering and grain filling in the savannas of WCA, as had been observed in cultivars of other maturity groups. Thus, a tremendous opportunity existed for improvement of the performance of the cultivars in the program by introgressing genes for improved tolerance to drought and *Striga* resistance. We recognized at the very early stages of the IITA extra-early maize improvement program that several genes governed the expression of drought tolerance in maize. Therefore, a major strategy of the program was to adopt various methods to identify maize inbred lines with tolerance to drought from diverse germplasm sources. Since 2007, various strategies have been used in the program for the genetic enhancement of the populations for drought tolerance at various testing sites in Nigeria. The focal point of the IITA extra-early-maturing maize program for improving adaptation to drought has been to screen maize inbred lines from diverse genetic backgrounds for tolerance to drought under managed moisture stress at Ikenne (Supplemental Table S1). The soil at the Ikenne experiment station is classified as eutric nitrosol (Soil Survey Staff, 1999), and the research fields are flat and uniform and characterized by high water-holding capacity. A sprinkler irrigation system was used to apply 17 mm of water weekly to the maize crop during the first 3 wk of growth in the dry season. The maize plants therefore depended on stored water in the soil for growth and development. This strategy ensured that flowering and grain-filling periods coincided with occurrence of induced drought stress. Under the optimal conditions at Ikenne, the plants were irrigated throughout the growing period using the sprinkler irrigation system, as described by Badu-Apraku et al. (2013a, 2017b). The trials were also evaluated under optimal conditions at Mokwa and Zaria (high-yield environments) in Nigeria to assess the yield of the cultivars. At Bagauda (characterized by terminal drought), the cultivars were exposed to drought stress that occurred from flowering until physiological maturity.

Badu-Apraku and Fakorede (2017) described in detail the strategies adopted to enhance cultivar resistance to *Striga* and tolerance to low N. Briefly, promising drought-tolerant, extraearly inbred lines selected for the development of the cultivars evaluated in the present genetic gain study were also screened for *Striga* resistance under artificial *Striga* infestation at Mokwa and Abuja. Drought-tolerant and *Striga*-resistant inbred lines also possessed tolerance to low N, even though they had not been specifically selected for tolerance to low N (at 30–40 kg N ha^−1^).

By 2007, extra-early inbreds and hybrids that possessed genes for tolerance to drought during flowering and grain-filling periods, and which were also capable of escaping drought (characteristic of extra-early-maturing cultivars) and had low-N tolerance genes, had been identified (Badu-Apraku and Fakorede, 2017). A program was therefore commenced in 2011 to generate extra-early cultivars possessing genes for tolerance to drought. Towards this end, tolerance to drought and low N in the extra-early white (TZEE-W Pop STR C_5_) and the extra-early yellow (TZEE-Y Pop STR C_5_) *Striga*-resistant source populations was improved by introgressing drought and low-N tolerance genes from 19 white and 20 yellow extra-early inbred lines with elevated levels of tolerance to drought and/or low N (Badu-Apraku and Fakorede, 2017). Two hundred testcrosses generated from crosses, which involved each population and outstanding inbreds with enhanced drought tolerance, were evaluated at Ikenne under induced drought stress during the 2011–2012 dry season. The top-performing 25% testcrosses from each source population were selected and recombined to reconstitute each population. This was followed by recombination of the top 10 testcrosses of each population to form experimental cultivars that were designated as ‘2012 TZEE-W DT STR C_5_’ and ‘2012 TZEE-Y DT STR C_5_’. A total of 56 extra-early-maturity maize cultivars from the three breeding periods (1995–2000, 2001–2006, and 2007–2012), possessing enhanced drought tolerance and *Striga* resistance, were used for the present study (Supplemental Table S2).

### Field Evaluation and Data Collection

The extra-early cultivars were evaluated under six induced or terminal-drought environments in Nigeria and Ghana and 17 optimal environments in Ghana, Republic of Benin, and Nigeria, from 2013 to 2016 (Supplemental Table S3). The drought trials at Ikenne were planted during the dry season, and 17 mm of water was supplied to the plots weekly using the sprinkler irrigation system. To create induced drought stress at this location, the drought trials were irrigated for only the first 21 d after planting, causing the maize plants to rely on residual moisture in the soil for growth and development. In contrast, terminal drought was achieved by delaying the planting of the trials such that the occurrence of drought stress coincided with 1 to 2 wk before flowering. Optimal environments used in the present study refer to environments where water and N were adequate for plant growth and development. An eight-by-seven lattice design, with three replications, was adopted for the trial. Each experimental unit comprised two 4-m-long rows, with inter-row spacing of 75 cm and a spacing of 40 cm between plants within rows. Initially, three seeds were planted per hill, and 2 wk after planting (2 WAP), thinning was done to two seedlings per hill to obtain a final population density of 66,666 plants ha^−1^. Basal fertilizer (60 kg each of N, P and K ha^−1^) was applied to the managed drought stress experiments during planting, whereas 60 kg N ha^−1^ was top-dressed at 2 WAP. However, for terminal drought and rainfed environments, basal fertilizer application rates were 60 kg ha^−1^ each of N, P and K at 2 WAP and 60 kg of N ha^−1^ at 4 WAP. Crop management practices were similar for both drought stress and rainfed experiments. Weeds were controlled manually, as well as through the use of herbicides, as needed.

Data were recorded on the measured traits as described in detail by Badu-Apraku et al. (2015b). Briefly, in the drought-stressed and rainfed plots, days to 50% anthesis (DA) and days to 50% silking (DS) were recorded as the number of days from planting to when 50% of plants per plot had started shedding pollen or extruding silks, respectively. Anthesis–silking interval (ASI) was computed as the difference between DS and DA. Plant height (PHT) and EHT were measured as the length from the base of the plant to the first tassel branch and the upper ear node, respectively. Root lodging (RL) was estimated as the percentage of plants leaning >30° from the vertical, whereas stalk lodging (SL) was computed as percentage of plants with broken stalks at or below the highest ear node. Plant aspect (PASP) was rated on a scale of 1 to 9 based on plant type, where 1 = excellent and 9 = poor. Ear aspect (EASP) was scored on a scale of 1 to 9, where 1 = clean, uniform, large, and well-filled ears and 9 = ears with undesirable features. Husk cover (HUSK) was rated on a scale of 1 to 5, where 1 = husks tightly arranged and extended beyond the ear tip and 5 = ear tips exposed. The EPP was determined by dividing the total number of ears harvested by the number of plants in the plot at harvest. In addition, stay-green characteristic (STGR) was scored for the drought-stressed plots at 70 d after planting on a scale of 1 to 9, where 1 represented plants with almost all leaves green, and 9 indicated plants with virtually all leaves dead. Grain yield for drought trials was adjusted to 150 g kg^−1^ moisture and estimated from the shelled grain weight. In the rainfed experiments, grain yield was determined from ear weight using 80% shelling percentage, adjusted to moisture content of 150 g kg^−1^.

### Statistical Analyses

Observations recorded on plot means for grain yield and other agronomic traits were subjected to ANOVA for drought stress and rainfed environments separately using PROC GLM statement of SAS 9.3 (SAS Institute, 2011). The environments were regarded as the location–year combinations in the combined ANOVA. The environments, replicates within environment, and blocks within replicates of each experiment were treated as random effects, whereas the entries were considered fixed effects. Means of the 56 cultivars for each variable were regressed on the year when the cultivar was developed to estimate gain per year for the respective traits. The means of grain yield and other traits of the maize cultivars were used as dependent variables, and regressed on the year of breeding, as the independent variable to obtain the linear regression coefficient (*b* value) under drought stress and rainfed environments. The relative genetic gain per year was estimated as the *b* value divided by the intercept and multiplied by 100 (Badu-Apraku et al., 2009). Similarly, the yield gain per period was computed by regressing mean grain yield of cultivars on the respective periods of development. Annual yield gains for cultivars of each of the breeding period were also computed following a similar procedure. The Excel software in the Microsoft Office Suite 2007 was used for the regression analysis, as well as for the estimation of the parameters and the graphical display of the regression lines. Correlation coefficients between grain yield and other measured traits of maize cultivars were computed for drought stress and rainfed growing conditions using SAS version 9.3 (SAS Institute, 2011). To facilitate the estimation of variance components, cultivars were treated as a random factor in this context. Variance components were computed using the restricted maximum likelihood (REML) option in PROC MIXED command (SAS Institute, 2011). The estimates of broad-sense heritability (*H*^2^) for grain yield were computed for each environment, and all the environments included in the present study revealed an *H*^2^ value of 0.30 (Supplemental Table S2). The *H*^2^ of grain yield and other traits were estimated as follows:

$$H^{2}=\frac{\sigma_{g}^{2}}{\sigma_{g}^{2}+\frac{\sigma_{e}^{2}}{r}}$$

where σ_g_^2^ is the variance attributable to genotypic effects, σ^2^_e_ is experimental error variance; and *r* is the number of replicates within each environment (Fehr, 1991).

Repeatability estimates of the traits (Falconer and Mackay, 1996) across environments were calculated on a cultivar-mean basis as follows:

$$R=\frac{\sigma_{g}^{2}}{\sigma_{g}^{2}+\frac{\sigma_{ge}^{2}}{e}+\frac{\sigma^{2}}{re}}$$

where *e* is the number of environments; σ^2^_ge_ is the component of variance attributable to cultivar × environment interaction (GEI), and σ^2^ is the error variance.

Stepwise regression analysis and sequential path diagrams were used to show the cause-and-effect relationships among traits in the present study. SPSS (SPSS, 2007) was used for the stepwise regression analyses to obtain information on the path coefficients and the causal relationships required for the path diagrams. Following the method proposed by Mohammadi et al. (2003), the predictor traits were organized into first, second, and third order, based on their contributions to the total variation in grain yield, with minimized multicolinearity (Badu-Apraku et al., 2014; Talabi et al., 2017). To perform the stepwise regression analysis, grain yield was regressed on measured traits to identify traits with significant contributions to the total variation in grain yield at *P* ≤ 0.05, which were categorized as first-order traits. The first-order traits thereafter were each regressed on other traits that were not in the first-order category, to identify traits with significant contributions to grain yield through the first-order traits. These traits were classified as second-order traits. The same procedure was repeated to identify third-order trait(s), and so on. The path coefficients were obtained from the standardized *b* values of the stepwise regression analysis (Badu-Apraku et al., 2014; Talabi et al., 2017). The significance of the path coefficients was tested using the SEs at the 0.05 probability level, with only traits having significant path coefficients retained in each order.

A selection index for drought tolerance, which incorporated grain yield of the cultivars under drought, along with the expression of traits such as ASI, PASP, EASP, STGR, and EPP, was used to characterize the extra-early maize cultivars as drought tolerant and drought sensitive (Oyekunle and Badu-Apraku, 2012). The effect of different scales was minimized by standardizing each parameter using a mean and SD of zero and one, respectively. Thus, a cultivar characterized by a positive value was considered drought tolerant, whereas the drought-sensitive cultivars were those with negative values. The selection index was calculated as follows:

Selection index = [(2 × yield) + EPP − ASI − PASP − EASP − STGR]

Based on this characterization, 35 cultivars (top 25, middle five, and worst five genotypes) were selected for stability analysis. The additive main effects and multiplicative interaction (AMMI) analysis was adopted to investigate the relationships among cultivars, environments, and GEI components of the yield of the selected 35 extra-early cultivars. The AMMI model partitioned GEI into several interaction principal component axes (IPCAs) through principal component analysis (Gauch and Zobel, 1988; Zobel et al., 1988; Crossa, 1990). The AMMI analysis was performed using the genotype main effect plus GEI (GGE) biplot (Yan, 2001a, 2001b), and the AMMI model equation used was that reported by Sadeghi et al. (2011). The AMMI biplot provided information on the performance and stability of the selected cultivars across drought and rainfed environments.

## RESULTS

### Analysis of Variance for Grain Yield and Other Traits and Yield Gains

Results of combined ANOVA for grain yield and other measured traits under contrasting drought and rainfed environments (Table 1) revealed significant environment, period, cultivar (period), cultivar (period) × environment interaction, and period × environment interaction mean squares for all measured traits, except period mean square for ASI, and environment × period mean square for DS, EHT, RL, and EPP under drought conditions. Grain yield varied from 1.19 to 1.54 Mg ha^−1^ for cultivars of Period 1 and Period 3 under drought, respectively, which corresponded with an overall annual yield gain of 3.28% (Tables 2 and 3). Annual yield gains of 0.0480, 0.0500, and 0.0002 Mg ha^−1^ were obtained under drought for cultivars developed during Periods 1, 2, and 3, respectively, whereas the gain in grain yield per period across the 56 cultivars was 0.17 Mg ha^−1^. Grain yield of cultivars ranged from 3.30 Mg ha^−1^ for Period 1 cultivars to 4.06 Mg ha^−1^ for Period 3 cultivars under rainfed conditions, which translated to an annual genetic gain of 2.25%. Under rainfed environments, cultivars of Period 1 showed an annual yield gain of 0.12 Mg ha^−1^, whereas the gains in grain yield obtained for Period 2 and Period 3 cultivars were 0.022 and 0.014 Mg ha^−1^, respectively. The yield gain per period of the 56 cultivars was 0.38 Mg ha^−1^ across rainfed environments. The realized annual increase in grain yield was 0.034 and 0.068 Mg ha^−1^ under drought stress and rainfed environments, respectively. The significant yield increase from Period 1 to Period 3 under drought stress and rainfed environments was associated with prolonged flowering period, increase in EHT and PHT, and improvement in SL resistance, EASP, and PASP. Other characters that accompanied the significant yield improvement under drought conditions included prolonged STGR and increased EPP (Table 3).

Under drought stress, positive and significant *b* values (gain per year) were obtained for grain yield, DA, DS, PHT, EHT, and EPP, whereas significant negative *b* values were observed for SL, PASP, EASP, and STGR. The same set of traits showed similar trends under rainfed environments, except EPP, for which no significant gain was obtained; STGR was not measured under rainfed environments (Table 3).

**Table 1:** Mean squares for grain yield and other agronomic traits of extra-early maize cultivars of three breeding periods evaluated under drought stress in six environments and under rainfed conditions in 17 environments in Nigeria, Benin, and Ghana from 2013 to 2016.

Entry	df	Grain yield	Days to anthesis	Days to silk	Anthesis-silking interval	Plant height	Ear height	Root lodging	Stalk lodging	Husk cover†	Plant aspect‡	Ear aspect§	Ear rot	Ears per plant	Stay green¶
		Mg ha~^1^	____________ d ________________			____________ cm ________________		____________ % ________________					%		
**Drought environments**															
Environment (E)	5	122,796,029**	4,015.6**	3,839.1**	100.0**	53,250.1**	12,167.6**	2,555.2**	1,057.7**	479.9**	775.5**	505.9**	4,258.1**	8.35**	594.1**
Block (E × replicate)	108	582,011**	5.1**	9.1**	2.4**	650.4**	266.2**	13.1**	21.1**	0.8**	0.9**	0.9**	6.6**	0.03**	1.3**
Replicate (E)	12	798,912**	26.4**	31.8**	1.5	1,247.4**	426.4**	39.2**	19.7**	1.4**	1.8**	2.8**	14.3**	0.02	2.8**
Era	2	10,230,293**	163.5**	128.6**	2.1	4,712.2**	1,604.7**	54.0**	129.8**	3.1**	20.7**	16.9**	27.2**	0.19**	7.5**
Cultivar (period)	53	787,274**	26.2**	31.1**	2.3**	801.2**	363.5**	12.9**	31.1**	0.8**	1.8**	1.3**	19.5**	0.03**	1.2**
E × cultivar (period)	265	250,476**	5.3**	6.1**	2.1**	268.9**	138.7*’	11.1**	16.0**	0.7**	0.9**	0.7**	14.2**	0.02**	0.9**
E × period	10	574,222**	7.4**	5.7	3.4*	631.0**	129.4	10.4	20.7**	3.1**	1.2**	1.4**	33.1**	0.01	2.6**
Error	548	131,885	2.3	3.3	1.4	190.9	113.2	7.8	8.7	0.2	0.4	0.3	5.2	0.01	0.4
Repeatability		0.78	0.84	0.84	0.09	0.74	0.71	0.26	0.54	0.14	0.67	0.63	0.28	0.42	0.38
**Rainfed environments**															
Environment (E)	16	12,755,930**	1,962.8**	2,574.8**	134.8**	97,075.2**	54,264.3**	6,919.2**	33,292.6**	46.6**	75.5**	57.7**	657.5**	2.00**	-
Block (E × replicate)	306	830,616**	4.5**	5.3**	1.1**	286.1**	216.1**	34.2**	84.3**	0.2**	0.6*	0.4**	2.7**	0.01**	-
Replicate (E)	34	4,759,476**	22.8**	23.0**	1.1	1,118.4**	673.4**	200.1**	497.5**	0.4**	0.8*	0.8**	4.6**	0.01**	-
Era	2	124,088,801**	743.1**	608.2**	5.4**	14,815.0**	10,611.3**	100.8**	499.6**	3.0**	13.5**	31.2**	6.8**	0.10**	-
Cultivar (period)	53	8,815,082**	79.9**	91.9**	1.3**	1,309.9**	974.6**	59.7**	242.1**	0.7**	2.1**	2.1**	4.4**	0.01**	-
E × cultivar (period)	848	698,835**	3.7**	4.1**	0.9**	220.0**	154.5**	27.5**	80.9**	0.2**	0.6**	0.2**	1.9**	0.01*	-
E × period	32	1,142,530**	7.1**	8.0**	2.0**	585.8**	330.6**	35.6**	133.3**	0.2*	0.8**	0.5**	3.1**	0.01*	-
Error	1,564	360,244	1.9	2.2	0.8	144.4	127.2	19.6	38.7	0.2	0.5	0.2	1.4	0.01	-
Repeatability		0.95	0.97	0.96	0.39	0.89	0.89	0.58	0.69	0.76	0.78	0.93	0.56	0.44	-

*,** Significant at 0.05 and 0.01 probability levels respectively.

† Husk cover was scored on a scale of 1 to 9, where 1 = husks tightly arranged and extended beyond the ear tip, and 9 = ear tips exposed.

‡ Plant aspect was recorded on a scale of 1 to 9 based on plant type, where 1 = excellent and 9 = poor.

§ Ear aspect rated on a scale of 1 to 9, where 1 = clean, uniform, large, and well-filled ears, and 9 = ears with undesirable features.

¶ Stay-green characteristic scored on a scale of 1 to 9, where 1 represented plants with almost all leaves green, and 9 indicated plants with virtually all leaves dead.

**Table 2:** Means ± SE for grain yield and other agronomic traits of extra-early maize cultivars of three breeding periods evaluated under drought stress in six environments and under rainfed growing conditions in 17 environments in Nigeria, Benin, and Ghana from 2013 to 2016.

Trait	Period	No. of cultivars	Drought conditions	Rainfed conditions
Grain yield (Mg ha^−1^)	1995–2000	14	1.190 ± 0.0512	3.296 ± 0.1214
	2001–2006	17	1.353 ± 0.0733	3.674 ± 0.1000
	2007–2012	25	1.538 ± 0.0361	4.056 ± 0.1218
Days to anthesis	1995–2000	14	53 ± 0.33	51 ± 0.43
	2001–2006	17	54 ± 0.39	53 ± 0.35
	2007–2012	25	54 ± 0.21	53 ± 0.27
Days to silking	1995–2000	14	55 ± 0.38	53 ± 0.45
	2001–2006	17	57 ± 0.44	54 ± 0.37
	2007–2012	25	56 ± 0.21	54 ± 0.28
Anthesis silking interval (d)	1995–2000	14	3 ± 0.12	2 ± 0.06
	2001–2006	17	3 ± 0.11	2 ± 0.04
	2007–2012	25	3 ± 0.06	2 ± 0.03
Plant height (cm)	1995–2000	14	147 ± 1.49	167 ± 1.58
	2001–2006	17	152 ± 1.95	173 ± 1.57
	2007–2012	25	154 ± 1.53	175 ± 1.22
Ear height (cm)	1995–2000	14	66 ± 1.13	79 ± 1.54
	2001–2006	17	69 ± 1.39	84 ± 1.16
	2007–2012	25	70 ± 0.92	85 ± 1.03
Root lodging (%)	1995–2000	14	3.6 ± 0.23	5.5 ± 0.32
	2001–2006	17	4.1 ± 0.27	5.4 ± 0.20
	2007–2012	25	3.3 ± 0.17	4.8 ± 0.28
Stalk lodging (%)	1995–2000	14	5.8 ± 0.42	11.6 ± 0.63
	2001–2006	17	6.1 ± 0.29	11.2 ± 0.54
	2007–2012	25	4.8 ± 0.29	9.8 ± 0.53
Husk cover†	1995–2000	14	3.0 ± 0.05	2.1 ± 0.03
	2001–2006	17	2.8 ± 0.06	2.0 ± 0.04
	2007–2012	25	2.8 ± 0.05	2.0 ± 0.02
Plant aspect‡	1995–2000	14	4.4 ± 0.10	2.5 ± 0.05
	2001–2006	17	4.0 ± 0.08	2.4 ± 0.04
	2007–2012	25	3.9 ± 0.07	2.3 ± 0.06
Ear aspect§	1995–2000	14	3.7 ± 0.07	2.8 ± 0.07
	2001–2006	17	3.5 ± 0.09	2.6 ± 0.05
	2007–2012	25	3.3 ± 0.05	2.4 ± 0.06
Ear rot (%)	1995–2000	14	4.5 ± 0.21	1.8 ± 0.09
	2001–2006	17	3.9 ± 0.34	1.7 ± 0.07
	2007–2012	25	4.5 ± 0.26	1.7 ± 0.06
Stay-green characteristic¶	1995–2000	14	4.2 ± 0.08	–
	2001–2006	17	3.9 ± 0.08	–
	2007–2012	25	3.9 ± 0.07	–
Ears per plant	1995–2000	14	0.7 ± 0.011	0.9 ± 0.0044
	2001–2006	17	0.8 ± 0.013	0.9 ± 0.0038
	2007–2012	25	0.8 ± 0.008	0.9 ± 0.0039

† Husk cover was scored on a scale of 1 to 9, where 1 = husks tightly arranged and extended beyond the ear tip, and 9 = ear tips exposed.

‡ Plant aspect was recorded on a scale of 1 to 9 based on plant type, where 1 = excellent and 9 = poor.

§ Ear aspect rated on a scale of 1 to 9, where 1 = clean, uniform, large, and well-filled ears, and 9 = ears with undesirable features.

¶ Stay-green characteristic scored on a scale of 1 to 9, where 1 represented plants with almost all leaves green, and 9 indicated plants with virtually all leaves dead.

**Table 3:** Relative genetic gains in grain yield and other agronomic traits of extra-early maize cultivars of three breeding periods across six drought and 17 rainfed research environments in Nigeria, Benin, and Ghana from 2013 to 2016.

Trait	Relative gain	*R*^2^	*a* (intercept)	*b* (linear regression coefficient)
	% yr^−1^			
**Drought stress environments**				
Grain yield (Mg ha^−1^)	3.28	0.358	1.03	0.034**
Days to anthesis	0.17	0.086	52.6	0.089*
Days to silk	0.16	0.076	55.3	0.087*
Anthesis silking interval (d)	−0.52	0.022	2.9	−0.015
Plant height (cm)	0.49	0.185	144	0.710**
Ear height (cm)	0.65	0.152	64	0.416**
Root lodging (%)	−1.05	0.045	4.1	−0.043
Stalk lodging (%)	−1.65	0.102	6.5	−0.107**
Husk cover†	−0.17	0.010	3.0	−0.005
Plant aspect‡	−0.91	0.171	4.5	−0.041**
Ear aspect§	−1.51	0.315	4.0	−0.061**
Ears rot (%)	−0.23	0.001	4.4	−0.010
Stay-green characteristic¶	−0.72	0.121	4.3	−0.031**
Ears per plant	0.40	0.101	0.8	0.003*
**Rainfed environments**				
Grain yield (Mg ha^−1^)	2.25	0.361	3.017	0.068**
Days to anthesis	0.30	0.199	50.7	0.150**
Days to silk	0.20	0.108	52.8	0.107**
Anthesis silking interval (d)	−0.45	0.025	2.0	−0.009
Plant height (cm)	0.44	0.289	164.5	0.727**
Ear height (cm)	0.77	0.264	76.8	0.591**
Root lodging (%)	−1.09	0.065	5.9	−0.064
Stalk lodging (%)	−1.17	0.077	12.2	−0.143*
Husk cover†	−0.32	0.061	2.1	−0.007
Plant aspect‡	−1.52	0.217	2.6	−0.040**
Ear aspect§	−1.75	0.284	3.2	−0.055**
Ears rot (%)	−0.91	0.046	2.0	−0.018
Ears per plant	2.25	0.059	0.9	0.020

*,** Significant at 0.05 and 0.01 probability levels, respectively.

† Husk cover was scored on a scale of 1 to 9, where 1 = husks tightly arranged and extended beyond the ear tip, and 9 = ear tips exposed.

‡ Plant aspect was recorded on a scale of 1 to 9 based on plant type, where 1 = excellent and 9 = poor.

§ Ear aspect rated on a scale of 1 to 9, where 1 = clean, uniform, large, and well-filled ears, and 9 = ears with undesirable features.

¶ Stay-green characteristic scored on a scale of 1 to 9, where 1 represented plants with almost all leaves green, and 9 indicated plants with virtually all leaves dead.

**Fig. 1:**
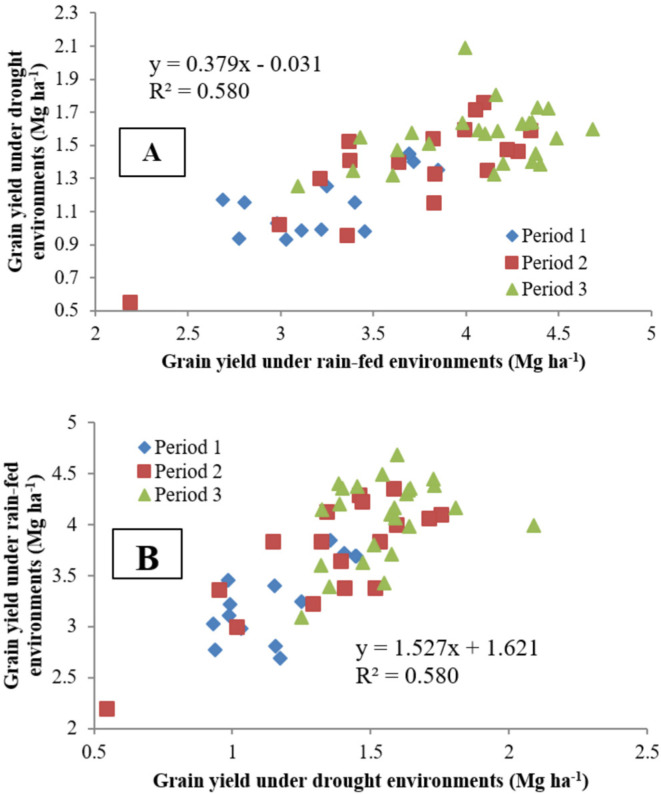
Regression of grain yield of extra-early maize cultivars of three breeding periods (A) under drought on yield performance under rainfed environments and (B) vice versa.

Regression of mean grain yield of the extra-early maize cultivars tested under drought conditions on mean grain yield under rainfed environments, and vice versa, clearly separated the maize cultivars into three distinct breeding periods (Fig. 1a and 1b). However, some cultivars from Period 2 produced yields comparable with those of Period 3 extra-early cultivars, whereas one Period 2 cultivar (TZEE-Y SR BC1 × 9450 STR S_6_ F_2_) produced yield lower than those of Period 1 cultivars. The extra-early Period 3 cultivars exhibited the most outstanding performance under both drought stress and rainfed environments. The grain yield of the cultivars under drought stress adequately predicted the yield performance of the cultivars under rainfed environments and vice versa (*R*^2^ = 58%, Fig. 1a and 1b).

## Interrelationships among Traits

**Fig. 2:**
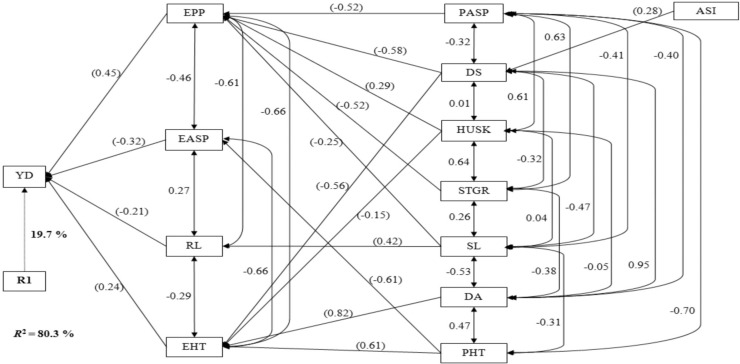
Path analysis model diagram showing causal relationships of measured traits of extra-early maize cultivars of three breeding periods, evaluated under drought stress at six environments in West Africa in 2013 to 2016. The bold value is the residual effect; values in parenthesis are direct path coefficients, whereas other values are correlation coefficients. R1, residual effects; ASI, anthesis–silking interval; DA, days to 50% anthesis; DS, days to 50% silking; EASP, ear aspect; EHT, ear height; EPP, ears per plant; HUSK, husk cover; PASP, plant aspect; PHT, plant height; RL, root lodging; SL, stalk lodging; STGR, stay green characteristics; YD, grain yield.

Under drought environments, the stepwise regression analysis identified EPP, EASP, RL, and EHT as first-order traits; these traits explained ~80% of the variability in grain yield (Fig. 2). Number of ears per plant had the largest path coefficient, whereas RL had the smallest path coefficient. The secondorder traits identified under drought included PASP, DS, HUSK, STGR, SL, DA, and PHT; each contributed to the variation in grain yield through one or two first-order traits. The highest indirect effect (0.82) was observed for DA through EHT, whereas the lowest indirect effect (−0.15) was obtained for HUSK through EHT. Five out of the seven second-order traits made significant contributions to grain yield through EPP, four through EHT, and one each through EASP and RL. Anthesis–silking interval was the only third-order trait identified under drought conditions in this study, which made significant contributions to grain yield through DA.

**Fig. 3:**
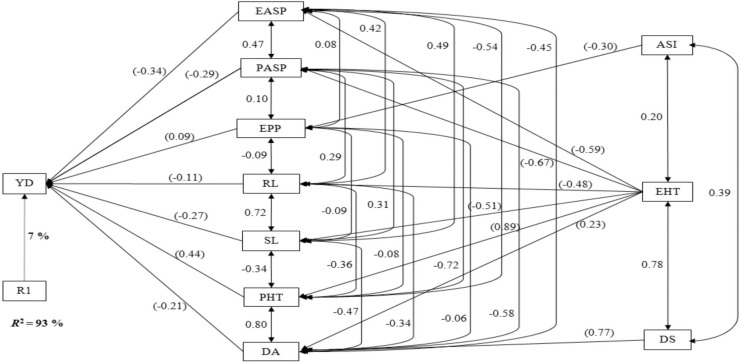
Path analysis model diagram showing causal relationships of measured traits of extra-early maize cultivars of three breeding periods, evaluated under rainfed conditions at 17 environments in West Africa in 2013 to 2014. The bold value is the residual effect; values in parenthesis are direct path coefficients, whereas other values are correlation coefficients. R1, residual effects; ASI, anthesis–silking interval; DA, days to 50% anthesis; DS, days to 50% silking; EASP, ear aspect; EHT, ear height; EPP, ears per plant; HUSK, husk cover; PASP, plant aspect; PHT, plant height; RL, root lodging; SL, stalk lodging; STGR, stay green characteristics; YD, grain yield.

Under rainfed environments, stepwise regression analysis classified seven traits (EPP, EASP, PASP, RL, SL, PHT, and DA) as the first-order traits (Fig. 3). These traits together contributed ~93% to the total variation in grain yield. Five of the traits contributing directly to grain yield showed negative effect, whereas two of the traits had positive effects. The largest direct contribution to grain yield was that of PHT (0.44), whereas the smallest contribution was that of EPP (0.09). Second-order traits identified under rainfed environments were ASI, EHT, and DS. Although EHT made significant contributions to grain yield through six first-order traits, ASI and DS each contributed through only one of the first-order traits.

### Performance and Stability of Extra-Early Maize Cultivars

**Fig. 4:**
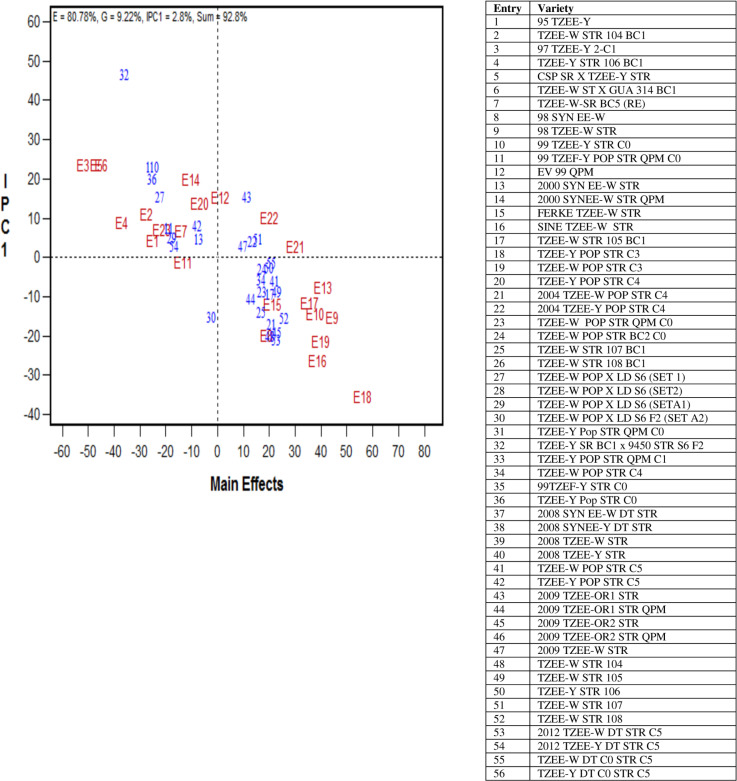
Mean performance and stability of selected 35 extra-early maturing maize cultivars of three breeding periods in terms of grain yield as measured by principal components across 23 drought and rainfed environments in West Africa between 2013 and 2016. E1 = Ikenne, drought, 2013; E2 = Bagauda, drought, 2013; E3 = Dusu, drought, 2013; E4 = Kpeve, drought, 2014; E5 = Ikenne, drought, 2014; E6 = Ikenne, drought, 2015; E7 = Ikenne, rainfed, 2013; E8 = Ife, high N, 2013; E9 = Zaria, rainfed, 2013; E10 = Mokwa, high N, 2013; E11 = Ina, rainfed, 2013; E12 = Angaradebou, rainfed, 2013; E13 = Maini-Hari, rainfed, 2013; E14 = Nyankpala, rainfed, 2013; E15 = Ikenne, rainfed, 2014; E16 = Ife, high N, 2014; E17 = Mokwa, high N, 2014; E18 = Zaria, rainfed, 2014; E19 = Bagauda, rainfed, 2014; E20 = Ina, rainfed, 2014; E21 = Angaradebou, rainfed, 2014; E22 = Manga, rainfed, 2014; E23 = Fumesua, rainfed, 2014.

The AMMI biplot for grain yield clearly depicted the performance of the selected 35 extra-early-maturing maize cultivars of the three breeding periods and stability across drought and rainfed environments (Fig. 4). The grand mean of grain yield was represented by the vertical dotted line, whereas the IPCA 1 value of zero was represented by the horizontal dotted line (*y* ordinate). The stable cultivars were those placed close to the horizontal line, with little interaction with the environments, whereas the less stable cultivars were those farther from the horizontal line. The high-yielding cultivars were placed to the right of the grand mean line, and the farther such cultivars were from the grand mean, the greater their grain yield. Across drought and rainfed environments, the percentage contributions of environment, cultivar, and IPCA 1 to the total variation in grain yield sum of squares were 80.78, 9.22, and 2.8, respectively. The 84.6% of the grain yield sum of squares captured by AMMI analysis was a clear indication that the biplot was effective in decomposing the GEI across drought stress and rainfed environments (Fig. 4). Cultivars ‘2004 TZEE-Y Pop STR C_4_’ and ‘TZEE-W Pop STR BC_2_ C_0_’ of Period 2 and ‘2009 TZEE-W STR’, ‘TZEE-Y STR 106’, ‘TZEE-W STR 107’, and ‘TZEE-W DT C_0_ STR C_5_’ of Period 3 were the most productive and stable relative to grain yield across drought and rainfed environments. Cultivar ‘2009 TZEE-OR_1_ STR’ yielded more than the mean grain yield but was adapted to highyield environments. A large number of cultivars, among which ‘TZEE-W STR 108’ was outstanding, were high yielding, with adaptation to low-yield environments.

## DISCUSSION

The significant cultivar means squares for all traits measured under drought and rainfed environments suggested that the cultivars were genetically distinct in the expression of these traits, which should facilitate the identification and selection of superior cultivars under the research conditions (i.e., drought and rainfed environments). Similarly, significant mean squares observed for environments for all measured traits under drought and rainfed environments were an indication that the environments were unique in their ability to discriminate among the cultivars under drought and rainfed environments. These findings corroborate results reported by Badu-Apraku et al. (2013a), who compared 50 early-maturing maize cultivars developed during three breeding eras under drought stress and optimal environments in WA. The significant cultivar × environment interactions detected for all the measured traits under drought and rainfed conditions suggested that the environments influenced the performance of the cultivars differentially and that multi-environment testing was desirable. However, this is inconsistent with the results of Badu-Apraku et al. (2013a), who observed lack of significant environment × era and environment × cultivar (era) effects for all the measured traits of early-maturing genotypes evaluated under drought conditions. The observed differences between the findings of Badu-Apraku et al. (2013a) and the results of the present study might have resulted from the fewer drought testing sites used in the former study.

An important objective of the present study was to investigate yield gains of 56 extra-early-maturing cultivars developed during three breeding periods under drought and rainfed environments. The extra-early cultivars showed an annual genetic gain of 3.28%, with a realized yield increase of 0.034 Mg ha^−1^ yr^−1^ under drought conditions, and 2.25% annual yield gain corresponding to an annual increase of 0.068 Mg ha^−1^ under rainfed conditions, which are greater than the yield gains obtained for the early-maturing cultivars reported by Badu-Apraku et al. (2013a), who reported annual yield gains of 1.1 (0.014 Mg ha^−1^) and 1.3% (0.040 Mg ha^−1^) under drought and well-watered conditions, respectively. The annual yield gain obtained for this set of extra-early maize cultivars under drought was also higher than the annual yield gains of 2.56% reported under artificial *Striga* infestation (Badu-Apraku et al., 2016), 2.14% under low soil N (Badu-Apraku et al., 2017a), and 2.72% under multiple-stress environments (Badu-Apraku et al., 2017b) for the same set of extra-early cultivars. Furthermore, the annual yield gain of 0.034 Mg ha^−1^ achieved under drought in the present study was greater than the gain of 0.029 Mg ha^−1^ obtained for CIMMYT’s ESA early-maturing OPVs (Masuka et al., 2017b) and was comparable with the annual yield gain of 0.042 Mg ha^−1^ reported for the CIMMYT’s ESA intermediate-late OPVs under random drought stress. The implications of the results obtained in the present study are that the extra-early OPVs had better responses to selection for improved grain yield and drought tolerance than the early and intermediate-late varieties tested under drought stress. Furthermore, the relative annual yield gain of 3.28% obtained for the extra-early cultivars under drought conditions was higher than the 2.25% achieved under rainfed environments in the present study. A plausible reason for this was that the emphasis of the breeding program was more on improvement in drought tolerance rather than performance of cultivars under rainfed environments. With the recent advances in molecular breeding techniques, marker-assisted selection and genomic selection schemes are presently being used to fast-track breeding processes and accelerate yield gains in our program. In addition to marker-assisted selection and genomic selection, several other strategies outlined by Masuka et al. (2017b) for increasing genetic gains in ESA breeding pipeline are being used in WCA under the Drought Tolerant Maize for Africa (DTMA)/Stress Tolerant Maize for Africa (STMA) Project for accelerating genetic gains. These include, among others, an increase in the size of the IITA maize breeding program to facilitate the use of higher selection intensity and an increase in the precision of selection to achieve higher heritability.

Meseka et al. (2006) indicated that drought-tolerant genotypes might be characterized using a selection index combining superior grain yield with desirable expression of PASP, EASP, and STGR, reduced ASI, and increased EPP under drought as well as high grain yield under optimal conditions. In the present study, the increased grain yields under drought and rainfed environments were associated with prolonged DA and DS, increases in EHT and PHT, and improvement in SL resistance, EASP, and PASP. In contrast, improved STGR and EPP accounted for yield gains only under drought environments. Gains achieved in grain yield associated with delayed leaf senescence during the breeding periods under drought may be attributed to a longer grain-filling period. The results of this study showed that the traits included in the selection index for characterizing drought tolerance were indeed effective in the development of superior cultivars under this stress factor. However, it was not effective in keeping constant the EHT and PHT and the flowering dates of the cultivars. Selection for improved tolerance to drought is usually conducted under drought stress, whereas field evaluations for drought tolerance are conducted under drought and rainfed environments. However, results of our studies have demonstrated repeatedly that outstanding cultivars identified under stress usually displayed outstanding performance under stress-free conditions (Badu-Apraku et al., 2013a). In this study, cultivar grain yield under drought adequately predicted yield performance of the cultivars under rainfed environments (*R*^2^ = 58%). The implication of this result is that the performance of the cultivars relative to grain yield under drought is a reliable indicator of expected yield performance of the cultivars under rainfed environments, and vice versa. Therefore, cultivars with outstanding performance under drought stress also display superior grain yield under rainfed conditions, and vice versa. Similar results were obtained by Badu-Apraku et al. (2013a) when early-maturing maize cultivars were evaluated under drought and optimal conditions.

Badu-Apraku et al. (2014) and Talabi et al. (2017) used the path coefficient analysis (Wright, 1921; Dewey and Lu, 1959) to quantify the contributions of various agronomic traits to the variation in grain yield. Of particular interest was the sequential path analysis, which allowed for categorization of traits into orders corresponding to the relative importance of the traits in explaining the variation in grain yield (Mohammadi et al., 2003). Under drought conditions, the identification of EPP, EASP, RL, and EHT as first-order traits implied that these traits could be useful for index selection for genetic enhancement of grain yield under drought stress. Of the four first-order traits, only EPP and EASP were among the traits included in the selection index (i.e., EASP, PASP, EPP, STGR, and ASI) along with grain yield for improvement of drought tolerance, emphasizing the importance of these traits when cultivars are subjected to drought stress. The categorization of PASP and STGR among the second-order traits was also an indication that these traits had potential value in selecting for drought tolerance. However, identification of ASI as a third-order trait in this study suggested that not only was this trait of least importance but also that it did not play a prominent role in justifying its use in the drought tolerance selection index in maize. The results of this study are inconsistent with the findings of Talabi et al. (2017), who identified ASI, EASP, PASP, STGR, and EPP as the primary traits directly responsible for the variability in grain yield of early-maturing full-sib progenies under drought stress. The difference in the findings may be explained by the differences in the genetic materials used for the present study; the cultivars evaluated in the present study were extra-early maturing, whereas Talabi et al. (2017) evaluated early-maturing full-sib progenies. This suggested that specific selection indices may be needed for the different types of genetic materials as well as maturity groups. Under rainfed environments, the identification of EASP, PASP, EPP, RL, SL, PHT, and DA as first-order traits implied that these traits were key in determining the variation observed in grain yield. Again, ASI was not in the first-order traits, as observed under drought but was among the second-order traits under rainfed conditions. The consistent identification of EASP and EPP as first-order traits under the contrasting environments confirmed their reliability for selection to improve grain yield across diverse environments. It is striking that Badu-Apraku et al. (2017a) placed EASP and PASP among the first-order traits under high- and low-N conditions for the same set of cultivars as used in this study. An important observation from the findings of several researchers (Badu-Apraku et al., 2011, 2014, 2017a; Talabi et al., 2017) is that EASP is a key trait accounting for the variation observed in grain yield under diverse stress conditions. Hence, EASP should be accorded the desired emphasis in selection programs designed to improve grain yield under contrasting environments to achieve concomitant improvement in tolerance to diverse stress environments.

Development of outstanding maize hybrids for adoption by small-scale farmers in SSA remains the most sustainable approach for increasing food security, alleviating poverty, and improving livelihoods in the subregion. The AMMI biplot identified the cultivars ‘2004 TZEE-Y Pop STR C_4_’ and ‘TZEE-W Pop STR BC_2_ C_0_’ from Period 2 and ‘2009 TZEE-W STR’, ‘TZEE-Y STR 106’, ‘TZEE-W STR 107’, and ‘TZEE-W DT C_0_ STR C_5_’ from Period 3 as highly productive and stable genotypes across drought and rainfed environments. These outstanding cultivars should be extensively tested in on-farm trials and commercialized for improving food self-sufficiency and farmers’ incomes in SSA. The cultivars ‘2009 TZEE-OR_1_ STR’, which was high yielding but adapted to high-yield environments, and ‘TZEE-W STR 108’, which was promising relative to grain yield but was adapted to low-yield environments, should be further tested for commercialization in the specific environments in which they displayed outstanding performance.

For more than two decades, early and extra-early maize cultivars have been developed for the savannas of SSA and extensively evaluated by IITA scientists in the subregion. Given the results of studies conducted under *Striga*-infested and *Striga*-free conditions, as well as those obtained from studies involving 50 early-maturing cultivars evaluated under drought, *Striga* infestation, and optimal conditions (Badu-Apraku et al., 2013b, 2014, 2017b), the conclusion was that early and extra-early maize responded favorably to selection under biotic and abiotic stresses encountered in SSA. Selection for drought and/or *Striga* tolerance or resistance has inadvertently led to improvement in the level of tolerance to low N, but not as much as the response to direct selection for low-N tolerance. Furthermore, selection under stress conditions results in improved performance of extra-early maize cultivars under stress-free environments. In addition, efforts at genetically enhancing maize for tolerance to drought in WCA have led to several conclusions that should guide breeders in SSA. The products of the research efforts include drought-tolerant early and extra-early populations, OPVs, inbred lines, and hybrids. Our experience has demonstrated unambiguously that the early and extra-early materials are capable of escaping drought and also possess genes for drought tolerance and can withstand drought stress that occurs randomly during the cropping season. Based on information on the DA and DS used as maturity indices, we have clearly established that there is tremendous genetic variability for the flowering traits in each maturity group. These flowering traits have been shown to have high heritability and significant negative phenotypic and genetic correlations with grain yield (Badu-Apraku and Fakorede, 2017). Therefore, early and extra-early maturities are under genetic control and are amenable to genetic enhancement and many maize improvement methods such as recurrent selection, pedigree selection, backcross breeding, double haploid, marker-assisted selection, and genomic selection.

## CONCLUSIONS

Based on the average annual rate of increase in grain yield under drought conditions (0.034 Mg ha^−1^) and under rainfed conditions (0.068 Mg ha^−1^), it can be concluded that considerable progress has been made during the last three decades in the genetic enhancement of extra-early maturing maize cultivars for drought tolerance in WCA. The availability of these extra-early cultivars is expected to contribute to improved food self-sufficiency, farmers’ incomes, and farmers’ livelihoods in SSA. The significant improvements in grain yield under drought and rainfed conditions were associated with prolonged DA and DS, increased EHT and PHT, and improvement in SL resistance, EASP, and PASP. In addition, delayed senescence and increased EPP accompanied significant improvement in productivity under drought. The EASP and EPP were consistently identified as highly reliable indirect selection criteria for improving grain yield through index selection under drought and rainfed environments. Highyielding and stable cultivars across all environments based on AMMI biplot included ‘2004 TZEE-Y Pop STR C_4_’ and ‘TZEE-W Pop STR BC_2_ C_0_’ of Period 2 and ‘2009 TZEE-W STR’, ‘TZEE-Y STR 106’, ‘TZEE-W STR 107’, and ‘TZEE-W DT C_0_ STR C_5_’ of Period 3. These cultivars could be commercialized to improve food self-sufficiency in SSA. Considerable improvement has been achieved in development and commercialization of drought-tolerant maize cultivars in the extra-early maturity group for the subregion.

## Abbreviations

AMMI: additive main effects and multiplicative interaction
ASI: anthesis–silking interval
DA: days to 50% anthesis
DS: days to 50% silking
EASP: ear aspect
EHT: ear height
EPP: number of ears per plant
ESA: Eastern and Southern Africa
GEI: genotype × environment interaction
HUSK: husk cover
IPCA: interaction principal component axis
MSV: *Maize streak virus*
OPV: open-pollinated variety
PASP: plant aspect
PHT: plant height
RL: root lodging
SSA: sub-Saharan Africa
SL: stalk lodging
STGR: stay-green characteristic
WAP: weeks after planting
WCA: West and Central Africa
