# Supplementary material for: Gains in Grain Yield of Extra-Early Maize during Three Breeding Periods under Drought and Rainfed Conditions
Source: Crop Sci. 2018 Aug 30;58(6):2399–412. doi: 10.2135/cropsci2018.03.0168 (PMC7680940; doi:10.2135/cropsci2018.03.0168)
Supplement: Supplementary file 1 [file CROPSCI-58-06-2399-s001.pdf]

Supplemental Table S1: Description of test locations used for the evaluation of the cultivars of three breeding periods under drought and rain-fed environments, 2013 to 2016.

Location	Agro ecological zone†	Latitude	Longitude	Altitude (m ASL)	Annual rainfall during growing season (mm)
Ikenne	RF	6^o87'N	3^o7'E	60	1500
Kadawa	SS	11^o45'N	8^o45'E	468.5	884
Bagauda	SS	12^o00'N	8^o22'E	580	884
Mokwa	SGS	9° 18'N	5° 4'E	457	1100
Zaria	NGS	11^o11'N	7^o38'E	640	1200

†NGS, Northern Guinea Savanna; RF, Rain forest zone; SGS, Southern Guinea savanna; SS, Sudan savanna.

Supplemental Table S2: Extra-early maize cultivars used in the study, their year of release/development and reactions to biotic and abiotic stresses.

Code	Cultivars	Year of development	Reactions to stresses		
			Drought	*Striga hermonthica*	Low N
1	95 TZEE-Y	1995	Susceptible	Susceptible	Susceptible
3	97 TZEE-Y 2-C_1	1997	Susceptible	Susceptible	Susceptible
5	CSP SR × TZEE-Y STR	1997	Susceptible	Susceptible	Susceptible
6	TZEE-W ST × GUA 314 BC_1	1997	Susceptible	Susceptible	Susceptible
7	TZEE-W-SR BC_5 (RE)	1997	Susceptible	Susceptible	Susceptible
8	98 SYN EE-W	1998	Susceptible	Susceptible	Tolerant
9	98 TZEE-W STR	1998	Tolerant	Susceptible	Susceptible
10	99 TZEE-Y STR C_0	1999	Susceptible	Susceptible	Susceptible
11	99 TZEF-Y Pop STR QPM C_0	1999	Susceptible	Susceptible	Susceptible
12	EV 99 QPM	1999	Susceptible	Susceptible	Susceptible
35	99 TZEF-Y STR C_0	1999	Susceptible	Susceptible	Susceptible
36	TZEE-Y Pop STR C_0	1999	Susceptible	Susceptible	Susceptible
13	2000 SYN EE-W STR	2000	Susceptible	Susceptible	Tolerant
14	2000 SYN EE-W STR QPM	2000	Tolerant	Susceptible	Susceptible
15	FERKE TZEE-W STR	2001	Susceptible	Resistant	Tolerant
16	SINE TZEE-W STR	2001	Susceptible	Susceptible	Susceptible
18	TZEE-Y Pop STR C_3	2001	Tolerant	Susceptible	Susceptible
19	TZEE-W Pop STR C_3	2002	Tolerant	Resistant	Tolerant
20	TZEE-Y Pop STR C_4	2002	Tolerant	Susceptible	Tolerant
21	2004 TZEE-W Pop STR C_4	2004	Tolerant	Tolerant	Tolerant
22	2004 TZEE-Y Pop STR C_4	2004	Tolerant	Tolerant	Tolerant
23	TZEE-W Pop STR QPM C_0	2004	Tolerant	Tolerant	Tolerant
24	TZEE-W Pop STR BC_2 C_0	2004	Tolerant	Tolerant	Tolerant
27	TZEE-W Pop × LD S_6 (SET 1)	2004	Tolerant	Tolerant	Tolerant
28	TZEE-W Pop × LD S_6 (SET2)	2004	Tolerant	Tolerant	Susceptible
29	TZEE-W Pop × LD S_6 (SETA1)	2004	Susceptible	Resistant	Tolerant
30	TZEE-W Pop × LD S_6 F_2 (SET A2)	2004	Susceptible	Susceptible	Tolerant
31	TZEE-Y Pop STR QPM C_0	2004	Susceptible	Susceptible	Susceptible
32	TZEE-Y SR BC_1 × 9450 STR S_6 F_2	2004	Susceptible	Susceptible	Susceptible
33	TZEE-Y Pop STR QPM C_1	2005	Susceptible	Tolerant	Tolerant
34	TZEE-W Pop STR C_4	2006	Tolerant	Tolerant	Tolerant
37	2008 SYN EE-W DT STR	2008	Tolerant	Susceptible	Tolerant
38	2008 SYNEE-Y DT STR	2008	Susceptible	Susceptible	Susceptible
39	2008 TZEE-W STR	2008	Tolerant	Tolerant	Tolerant
40	2008 TZEE-Y STR	2008	Susceptible	Susceptible	Susceptible
41	TZEE-W Pop STR C_5	2008	Tolerant	Resistant	Tolerant
42	TZEE-Y Pop STR C_5	2008	Tolerant	Susceptible	Susceptible
43	2009 TZEE-OR_1 STR	2009	Tolerant	Tolerant	Tolerant
44	2009 TZEE-OR_1 STR QPM	2009	Susceptible	Tolerant	Tolerant
45	2009 TZEE-OR_2 STR	2009	Tolerant	Resistant	Tolerant
46	2009 TZEE-OR_2 STR QPM	2009	Susceptible	Tolerant	Tolerant
47	2009 TZEE-W STR	2009	Susceptible	Tolerant	Susceptible
48	TZEE-W STR 104	2009	Tolerant	Resistant	Tolerant
49	TZEE-W STR 105	2009	Tolerant	Resistant	Tolerant
50	TZEE-Y STR 106	2009	Susceptible	Tolerant	Susceptible
51	TZEE-W STR 107	2009	Tolerant	Resistant	Tolerant
52	TZEE-W STR 108	2009	Tolerant	Resistant	Tolerant
2	TZEE-W STR 104 BC_1	2010	Susceptible	Resistant	Tolerant
4	TZEE-Y STR 106 BC_1	2010	Tolerant	Tolerant	Tolerant
17	TZEE-W STR 105 BC_1	2010	Tolerant	Resistant	Tolerant
25	TZEE-W STR 107 BC_1	2010	Tolerant	Tolerant	Tolerant
26	TZEE-W STR 108 BC_1	2010	Tolerant	Resistant	Tolerant
53	2012 TZEE-W DT STR C_5	2012	Tolerant	Resistant	Tolerant
54	2012 TZEE-Y DT STR C_5	2012	Susceptible	Tolerant	Susceptible
55	TZEE-W DT C_0 STR C_5	2012	Tolerant	Resistant	Tolerant
56	TZEE-Y DT C_0 STR C_5	2012	Tolerant	Susceptible	Tolerant

Supplemental Table S3. Environments, locations, research conditions and years of evaluation of extra-early maturing maize cultivars under drought-stress and rain-fed growing environments in West Africa.

Environment	Country	Location	Management	Year	Grain yield (Mg ha^{-1})	Heritability
1	Nigeria	Ikenne	Managed drought	2013/2014	2.438	0.67
2	Nigeria	Ikenne	Managed drought	2014/2015	0.760	0.71
3	Nigeria	Ikenne	Managed drought	2015/2016	1.046	0.73
4	Nigeria	Bagauda	Terminal drought	2013	2.237	0.57
5	Nigeria	Dusu	Terminal drought	2013	0.284	0.78
6	Ghana	Kpeve	Terminal drought	2014	1.649	0.65
7	Nigeria	Bagauda	Rain-fed	2014	4.421	0.76
8	Ghana	Fumesua	Rain-fed	2014	2.493	0.54
9	Benin	Ina	Rain-fed	2013	2.858	0.67
10	Benin	Ina	Rain-fed	2014	3.053	0.72
11	Nigeria	Ikenne	Rain-fed	2013	2.964	0.51
12	Nigeria	Ikenne	Rain-fed	2014	3.367	0.86
13	Nigeria	Mania	Rain-fed	2013	2.210	1.00
14	Ghana	Manga	Rain-fed	2013	3.447	0.53
15	Ghana	Nyankpala	Rain-fed	2013	2.975	0.34
16	Ghana	Nyankpala	Rain-fed	2014	3.441	0.47
17	Nigeria	Zaria	Rain-fed	2013	4.841	0.82
18	Nigeria	Zaria	Rain-fed	2014	5.670	0.91
19	Benin	Angaradebou	Rain-fed	2013	3.234	0.37
20	Benin	Angaradebou	Rain-fed	2014	3.793	0.61
